# Hologram Noise Model for Data Augmentation and Deep Learning

**DOI:** 10.3390/s24030948

**Published:** 2024-02-01

**Authors:** Dániel Terbe, László Orzó, Barbara Bicsák, Ákos Zarándy

**Affiliations:** HUN-REN Institute for Computer Science and Control (SZTAKI), 1111 Budapest, Hungary; terbed@sztaki.hu (D.T.); orzo.laszlo@sztaki.hu (L.O.);

**Keywords:** deep learning, neural networks, CNN, noise modeling, image augmentation, image processing, hologram

## Abstract

This paper introduces a noise augmentation technique designed to enhance the robustness of state-of-the-art (SOTA) deep learning models against degraded image quality, a common challenge in long-term recording systems. Our method, demonstrated through the classification of digital holographic images, utilizes a novel approach to synthesize and apply random colored noise, addressing the typically encountered correlated noise patterns in such images. Empirical results show that our technique not only maintains classification accuracy in high-quality images but also significantly improves it when given noisy inputs without increasing the training time. This advancement demonstrates the potential of our approach for augmenting data for deep learning models to perform effectively in production under varied and suboptimal conditions.

## 1. Introduction

The deep learning community has made remarkable progress in achieving state-of-the-art results on well-curated computer vision benchmark datasets [1,2,3,4,5]. Such datasets provide a consistent evaluation metric, allowing researchers to compare and innovate model architectures and training techniques. Nonetheless, bridging the gap between these benchmarks and the challenges posed by real-world scenarios remains an area that requires more exploration and attention [6,7]. While machine vision systems are typically trained and evaluated on high-quality image datasets, in practical applications, it cannot be presumed that images will maintain the same quality continuously during long measurements. Image quality, therefore, still remains a crucial yet frequently overlooked challenge in the design and implementation of these types of systems [8].

In this study, we tackle a common issue encountered during the deployment of neural networks, the potential alteration in the quality of input data over time, which can significantly compromise model performance if not addressed adequately [9,10,11]. We focus on the challenging task of classifying digital holographic microscope (DHM) images obtained from diluted water samples collected from waterworks. A notable aspect of this challenge is the limited availability of quality-degraded samples, which stands in contrast to the larger database of clean, standard samples. Our approach proposes a robust solution to maintaining high classification accuracy even when input data quality varies.

The objectives of our research are threefold:

- Analyzing the noise characteristics present in our hologram dataset.
- Proposing a sophisticated noise model based on empirical findings to generate realistic synthetic noise.
- Studying the effect of noise augmentation on neural network training.

In the context of a holographic microscope setup intended for extended (24 h) flow-through water sample monitoring, the clarity of samples can deteriorate over time due to factors such as contamination of the cuvette or the water sample itself. Predominantly, the system yields high-quality holograms, ensuring a database for neural network (NN) training populated by these uncontaminated samples. However, a decline in image quality can trigger a considerable drop in classification performance.

Although one solution is to enhance the robustness of the NN by amassing noisy samples, certain challenges still arise. Collecting sufficient training samples is challenging due to rare object classes and the infrequent occurrence of noisy samples, making the task cumbersome, expensive, and time-consuming. Instead, our study introduces a special method to handle noisy samples. By studying the noise present in holograms and crafting a comprehensive noise model based on empirical data, we can emulate realistic noise. This approach permits us to infuse synthetic random noise into clean samples in the dataset, facilitating immediate and effective augmentation for NN training. Consequently, the trained NN can classify both clean and noisy samples, demanding only uncontaminated standard samples for its training, as the noise information is integrated through the data augmentation process.

Several related studies predominantly focus on technical noise, such as photon noise and quantization noise, and coherent noise, such as speckle noise [12,13,14]. The authors in [8] have shown that deep neural networks are susceptible to white Gaussian noise. However, the impact of including low-quality images in the training set was not examined in their experiments, but it was later addressed in [10]. This paper analyzed the benefits of training neural networks using data affected by various types of noise, including Gaussian white noise and salt-and-pepper noise, for applications that need to deal with images of varying quality. Koziarski and Cyganek [7] evaluated the same types of noise in their study. They focused on two data-centric methods of dealing with noise: training with noise augmentation and denoising data in a preprocessing stage (with a denoising neural network). Their experimental results show that data augmentation is more beneficial for classification accuracy. Momeny et al., in [15], proposed a noise-robust convolutional neural network (NR-CNN) where a noise map layer is placed at the beginning of the neural network and used to detect impulse noise, missing image samples, packet loss in image transmission, damaged images, and tampered images. Yim and Son [16] proposed a two-column neural network utilizing both the original input and the denoised images to handle common noise types, like Gaussian white noise, speckle noise, and salt-and-pepper noise. The study by Shi et al. [17] presents a noise-fusion method (NFM) as a defense against adversarial attacks on DNN image classification models. This method involves adding white noise to both the model input at runtime and the training data during the training phase. The study demonstrated that adding noise not only defends against all three adversarial attacks but also improves the robustness of the corresponding models. Haekal et al. [18] conducted a study comparing the effects of Perlin noise- and Gaussian noise-based augmentation on the classification of X-ray images for lung cancer. Their findings indicate that noise-based augmentation can enhance the performance of deep learning in medical image classification with small datasets. Kim et al. [19] proposed a training paradigm for deep image recognition networks that generate clean-like features from any quality image via an invertible neural architecture, enhancing recognition tasks. Ceylan and Erer [20] explored a despeckling-based data augmentation approach to deep learning-based radar target classification, finding that despeckling-based data augmentation improves classification performance. Cai et al. [21] found that contrastive learning combined with convolutional neural networks improves radar signal intra-pulse modulation classification, outperforming other deep models and traditional methods in scenarios with Gaussian and impulsive noise-affected signals.

Unlike existing research, which primarily focuses on simple noise types, our study explores the complex scattering and interference-induced noise patterns of reconstructed hologram images that arise from capturing diluted water samples tainted with high concentrations of sub-micron particles. Consequently, we propose a method that enables the analysis, modeling, and synthesis of complex noise that can be used effectively during neural network training through an augmentation process.

The remainder of the paper is organized as follows: First, we elaborate on the noise types and present the available methods to analyze and construct intricate noise models. Next, we describe our measurement system, the collected dataset, and the details of neural network training. Finally, we present our results regarding hologram noise analysis, noise model construction, and NN training with noise model augmentation.

## 2. Materials and Methods

### 2.1. Noise Modeling

Noise, a ubiquitous element in recorded datasets, originates from various sources, such as environmental factors and system imperfections. Understanding and modeling noise is crucial to both comprehending its nature and devising mitigation strategies.

White noise is distinguished by its spatial independence, where the noise at each given data point (or pixel, in the case of images) is independent of its neighbors. For example, an archetypal instance of white noise in electronics and optics is shot noise. In electronics, it relates to the discrete nature of electric charge, while in optics, shot noise stems from the quantum characteristics of photons.

In contrast to white noise, colored noise showcases spatial correlations. The noise intensity at one data point can be influenced by those around it. Among the varieties of colored noise, “salt and pepper noise” [22] is frequently seen in digital imagery. This type of noise appears as sporadic pixels that stand in stark contrast to their surroundings, often manifesting as distinctly white or black points, resulting from glitches during data transmission. Another manifestation of colored noise is speckle noise, predominantly seen in imaging techniques such as ultrasound or coherent imaging. Speckle noise is a multiplicative noise that arises from random interference patterns, lending a grainy appearance to images. Pink noise, also known as “1/f noise” [23], is characterized by its frequency-dependent power spectrum, where the power decreases with the increase in frequency. This type of noise is often found in natural systems, including biological processes [24] and financial markets. Brown noise, or “$1/f^{2}$ noise” [25], is similar but has stronger correlation between adjacent points. Its power spectral density is inversely proportional to the square of the frequency, exhibiting a “$1/f^{2}$” relationship. This noise type can be generated through the cumulative sum or integration of white noise, a process which is fundamentally akin to a random walk in discrete-time settings or Brownian motion in continuous-time scenarios.

For this research, we collected a dataset of holographic images from dilute water samples. The observed noise may be due to water contamination caused by particles smaller than a micrometer (or/and the contamination of the cuvette), which results in complex scattering noise that exhibits 1/f pink noise-like characteristics (as we mentioned earlier, this is common in biosystems [24]).

In the following, we will provide more detailed mathematical and computational models for both white and colored noise.

#### 2.1.1. White Noise

White noise in the nexus of images, meaning that noise is uniformly distributed across the image and independent between any two pixels. The term “white” is an analogy to white light, which comprises all frequencies (or colors) of light combined. In the context of noise, “white” means that all spatial frequencies have equal intensity in its power spectral density. Mathematically, this is described as

(1) $$E\left[w\left(x,y\right)\right]=\mu_{x,y}=0$$

(2) $$E\left[w\left(x_{1},y_{1}\right)w\left(x_{2},y_{2}\right)\right]=\sigma_{w}^{2}\cdot \delta \left(x_{1}-x_{2},y_{1}-y_{2}\right)$$

where:

- E[.] represents the expected value operator.
- (x,y) are the spatial coordinates in the hologram.
- $\sigma_{w}$ is the standard deviation of white noise.
- δ is the 2D Dirac-delta function.

Equation (1) asserts that the expected value (or mean) of the noise at any point (x,y) is zero. The autocovariance defined by Equation (2) conveys that the noise at any two distinct spatial points is uncorrelated—except when the points coincide, i.e., when $x_{1}=x_{2}$ and $y_{1}=y_{2}$ –, since it is described by the delta function. The variance of the noise at each point is $\sigma_{w}^{2}$, and there is no relationship between the noise at any two distinct points.

Our computational model of white noise derived from our in-house dataset indicates that pixel intensities follow a normal distribution. Therefore, sampling the additive noise from a 2D normal distribution for each pixel is sufficient:

(3) $$w\left(x,y\right)∼N\left(0,\sigma_{w}^{2}\right)$$

where $N(0,\sigma_{w}^{2})$ denotes a normal distribution with mean 0 and variance $\sigma_{w}^{2}$.

Our selection of an additive noise model is grounded in the observation that noise characteristics of our dataset primarily emerge from a uniformly illuminated background. Given that the objects in our dataset are relatively small compared with this background, their individual perturbations have a minimal impact on the overall noise profile. This assumption allows us to focus on how background noise influences neural network training, rather than the minor variations caused by the objects themselves.

#### 2.1.2. Colored Noise

Colored noise describes a stochastic signal where spectral power varies across frequencies, unlike white noise, which remains constant. The term “colored” is an allusion to the fact that just as different colors of light have different frequencies, colored noise emphasizes certain frequencies over others. This results in various “colors” of noise, each characterized by its distinct spectral properties. In the realm of images or spatial data, colored noise implies the presence of correlations among pixels, leading to discernible patterns or structures in the noise (as mentioned earlier, some examples of noise color types are “pink” noise and “brown” noise).

Spatial correlations are encapsulated by the autocorrelation function (ACF). Autocorrelation is a measure of the similarity between values of a process at different spatial points, defined as the autocovariance normalized to the variance of the data (for example, the ACF of the previously discussed white noise is the Dirac-delta function).

For this study, we postulate that the noise present in our noisy holograms is best described using an exponentially decaying ACF ($e^{-\lambda x}$):

(4) E[c(x,y)]=0

(5) $$E\left[c\left(x_{1},y_{1}\right)c\left(x_{2},y_{2}\right)\right]=\sigma_{c}^{2}\exp\left(-\lambda \sqrt{{\left(x_{1}-x_{2}\right)}^{2}+{\left(y_{1}-y_{2}\right)}^{2}}\right)$$

where $\sigma_{c}^{2}$ represents the variance of colored noise and λ describes the spatial correlation length, which sets how quickly the noise correlations diminish as we move away from a specific point.

Modeling colored noise computationally is more complex because we must consider both the normal pixel intensity distribution and the exponential correlation. While the traditional method of synthesizing correlated noise involves the Cholesky decomposition of the covariance matrix (constructed using Equation (5)), this approach is computationally intensive for high-resolution images. Instead, we harness the Fourier space to generate spatially correlated noise. The Fourier transform of the 2D autocorrelation function (or the power spectral density) provides a spatial frequency representation of the noise (i.e., a Fourier kernel that represents the desired spatial noise properties). By multiplying this with the Fourier transform of white noise and then taking the inverse Fourier transform, we obtain spatially correlated noise. If the initial white noise had a normal distribution, then the resulting spatially correlated noise will also exhibit a normal distribution.

This approach is computationally efficient and easily adaptable to different noise models. By designing the appropriate Fourier kernel, various noise characteristics can be mimicked, allowing us to generate realistic noise that aligns with experimental observations.

To generate a hologram with spatially correlated noise, the following procedure can be adopted:

1. Create a white noise image with sampling from the standard normal distribution: w(x,y).
2. Compute the Fourier transform of the white noise image, denoted by F{w(x,y)}.
3. Multiply the result with the Fourier transform of the desired unnormalized ACF (which is, in our case, the exponential decay function) elementwise. Let us denote this product by $P(k_{x},k_{y})$, where $k_{x}$ and $k_{y}$ are the spatial frequency variables in the Fourier domain.
4. Compute the inverse Fourier transform of $P(k_{x},k_{y})$ to obtain the spatially correlated noise: c(x,y).
5. Add the synthesized noise (c(x,y)) to the original noise-free reconstructed hologram (x,y) elementwise.

In this study, our focus is on exponentially correlated noise, and the kernel was derived accordingly. However, this well-known methodology is general and can be adapted to various noise profiles as needed.

### 2.2. Microscope Setup and Database

An in-line holographic microscope system was used to capture hologram images. It operates with a wavelength of 405 nm. The camera used in the microscope has a pixel size of 2 μm, which provides an optimal balance between field of view and resolution. For the holograms, we used a matrix size of 2048 × 2048 pixels, allowing for comprehensive capture of the sample area. Our system produces background-subtracted holograms of dilute water samples in a flow-through cell. Contaminations on the flow-through cell walls and in the optical system are removed through the background subtraction process. If they are not significant, they will not considerably deteriorate the quality of the hologram. Once the raw hologram was captured, the objects in the hologram were digitally focused/reconstructed with the angular spectrum method [26] and then cropped with a 256 × 256 window around their centers. Holograms, when reconstructed, resulted in complex images expressed as (z=a+ib). We analyzed both the real (*a*) and imaginary (*b*) parts of these images and used them as inputs for the neural network.

**Figure 1:**
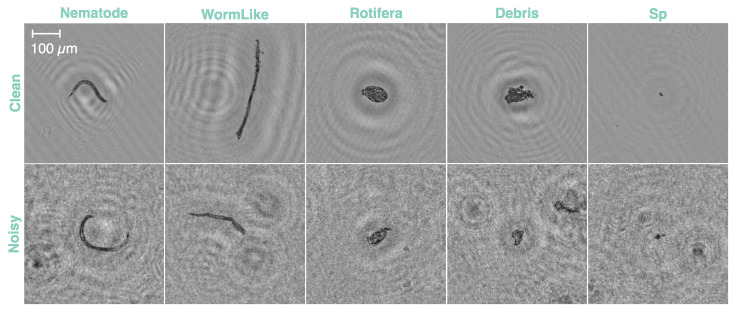
Examples for each class from the clean training dataset and the noisy test dataset. The scale bar is only denoted on the first image but applies to all other images.

In collaboration with waterworks, we obtained a real-life database to analyze water samples. The dataset used throughout this study includes 5 categories: *Nematode,* which is an elongated worm; *Rotifera*, which is a more round-looking worm; *WormLike*, which resembles an elongated worm, but it is an inanimate residue; *Sp*, which denotes small particles; and *Debris*, which contains inanimate remains of large-to-small sizes. Each class contains approximately 1000 clean samples. A clean sample and a noisy example for each category are shown in Figure 1. Collecting and labeling the database poses several challenges. Accumulating data from *Nematode* and *Rotifera* classes is demanding because these worms are not commonly found in such samples. Further, there are instances where even human evaluators may have difficulty in classifying the actual object. This inherent ambiguity in sample labeling naturally introduces some overlap or confusion between similar classes. For instance, as illustrated in Figure 1, elements from the *Rotifera* class visually resemble some elements in the *Debris* class. Additionally, the *Debris* class has a wide range of object sizes, making it possible for some elements to overlap with the *Sp* class.

As previously noted, gathering samples of worm classes is challenging, whereas there is an abundance of debris and small particles. The main problem to be solved in this specific use case is to accurately detect rarely occurring worms (that are either harmful or can indicate the contamination or malfunction of the filtration system) among numerous negative (innocuous) samples.

Regarding the noisy samples, we have fewer data available: approx. 183 nematode samples, 43 worm-like objects, 36 rotifera samples, approx. 1500 small-particle samples, and 1500 debris samples. Therefore, the noisy samples were only used for testing purposes, and they were not included in the training of the neural networks. A subset of the small-particle class (70 instances) was used for the construction of the noise model.

### 2.3. Neural Network and Training

In this work, our aim is to introduce an augmentation technique that can enhance the performance of any deep learning model in the case of noisy samples rather than proposing a specific neural network architecture. Thus, the utilized architecture has no great importance in this study. Nevertheless, in this section, we will provide a brief overview of the employed neural network architecture and describe the training process in more detail.

The deep learning framework of Pytorch 2.0 is used to build and train the neural networks. We split our clean dataset into training, validation, and test sets (65:20:15). The Adam optimizer is used to adjust the model parameters with a learning rate of 2 $\times 10^{-4}$ and a weight decay of 0.01. An early-stopping mechanism is implemented to halt training if validation does not improve during the last 10 epochs. Standard augmentations (rotation, horizontal flip, and vertical flip) are applied to avoid overfitting and achieve better generalization.

The input of the NN comprises the real and imaginary parts of the reconstructed hologram object (2-channel input). The architecture adopts a convolutional neural network framework, integrating skip connections reminiscent of a ResNet structure. It consists of 5 convolution blocks and a fully connected classification head with 2 layers. A convolution block comprises 2 convolutions with a kernel size of 3 × 3, batchnorm operations, and leaky ReLu activations. There are skip connections between convolution blocks. After the convolutional blocks, there is an adaptive average pooling layer that reduces the spatial dimensions to 2 × 2. The output is then flattened into a 512-element vector, which serves as the input for the fully connected head. The hidden layer has 256 neurons, and the output layer has 5 units—which is the number of classes. Before the linear layers, a dropout layer with a drop probability of 0.5 is utilized. After the first linear layer, a leaky ReLU activation function is applied, followed by a log softmax function after the output layer. Consequently, the network is trained by utilizing a negative log-likelihood loss (NLLLoss) function:

(6) $$L=-\sum_{c=1}^{N}w_{c}y_{c}l_{c}$$

where *N* represents the total number of classes, $w_{c}$ is the weight assigned for balancing classes, $y_{c}$ signifies the binary class label (1 for the correct class and 0 otherwise), and $l_{c}$ corresponds to the network’s output. As previously indicated, the terminal layer employs a LogSoftMax transformation:

(7) $$l_{c}=\log\left(\frac{e^{z_{c}}}{\sum_{i=1}^{N}e^{z_{i}}}\right)$$

where $z_{c}$ stands for the raw output (logits) from the network prior to the normalization layer depicted above. It is important to mention that this is equivalent to using cross-entropy loss with a softmax activation function in the output layer.

We train three identical neural networks with different training techniques:

1. *Base model*: Only trained on clean samples (without noise augmentations).
2. *White-augmented model*: Model trained with white noise augmentation.
3. *Color-augmented model*: Model trained with colored noise augmentation.

The noise augmentation operations are applied to the original clean samples with a 50% chance during training. Each model is trained on the exact same dataset. Although each model is only trained on clean samples, the augmentation process allows the neural network to learn the noise model, which in turn incorporates information from noisy samples.

## 3. Results

In this section, we present our findings regarding (1) the noise present in the (reconstructed) holograms of contaminated water samples and (2) the training of neural networks with and without noise augmentation.

### 3.1. Noise Analysis

We selected 70 noisy reconstructed holograms with dimensions of 256 × 256 for statistical analysis. The images contained only small objects (Sp) and featured consistent and homogeneous background noise. The reconstructed hologram objects were segmented from the images, ensuring that only the ambient noise was examined. Due to the high level of noise, the twin image of each segmented small object became visually indiscernible. Additionally, averaging across multiple independent images helps mitigate this effect, though ideally, the background should be analyzed without the presence of any object. Both the real and imaginary parts of the reconstructed hologram noise were analyzed.

**Figure 2:**
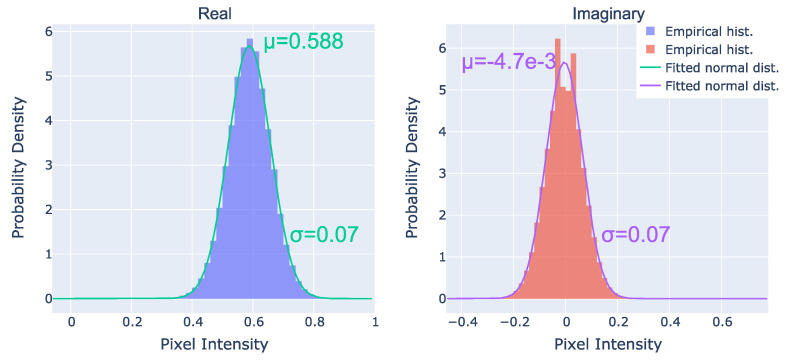
The pixel intensity distributions of the real and imaginary parts of the reconstructed hologram background noise. Statistics were computed from 70 noisy background images that had a size of 256 × 256.

First, we inspected the pixel intensity distribution of the noise. Both the real and the imaginary parts followed a normal distribution with a standard deviation of 0.07, as shown in Figure 2.

**Figure 3:**
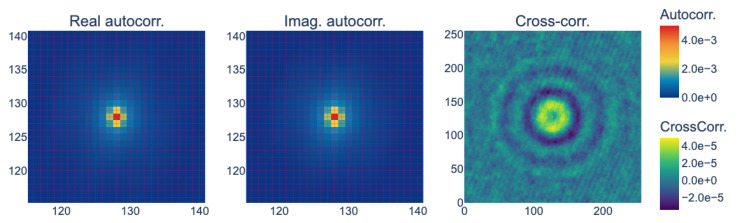
The averaged empirical 2D autocorrelation of the real and imaginary parts of the reconstructed hologram and their cross-correlation. The autocorrelation of the noise is not a Dirac-delta-like function—it has an isotropic spread—suggesting that the noise is not a simple uncorrelated white noise but rather a correlated colored noise. The average cross-correlation is significantly smaller, by two orders of magnitude.

The spatial characteristics were then investigated, particularly the unbiased and unnormalized autocorrelation of the noise and the cross-correlation between the real and imaginary components. The results suggest that the noise maintained some spatial correlation; see Figure 3. If the noise had been uncorrelated (white noise), the autocorrelation would have shown a single peak at the origin (i.e., it would only have had a zero-lag component). However, in this case, it had an isotropic spread, indicating spatial correlation. The cross-correlation between the real and imaginary parts was significantly lower than the autocorrelation. Hence, we can simplify the noise modeling and synthesis by assuming that the real and imaginary parts of the noise are independent. The weak correlation between these terms (which retains an intricate pattern) might be introduced by the propagation algorithm [26] while focusing on the objects.

**Figure 4:**
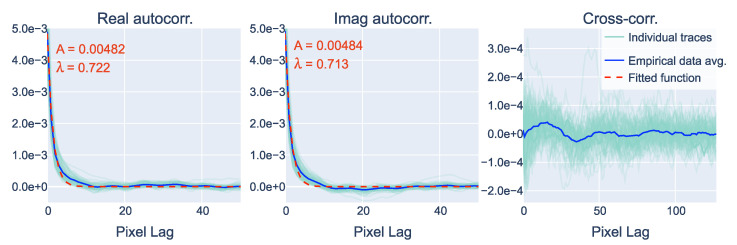
The middle horizontal cross-sections of the averaged 2D autocorrelation and cross-correlation data (Figure 3). An exponential function was fitted to the empirical autocorrelation data to model the noise properties.

Since the 2D autocorrelation of the noise is isotropic, we can describe it with a 1D function that represents its autocorrelation based on the distance from the zero-lag position. We can express the noise properties with 1D empirical autocorrelation by taking the middle cross-section of the 2D autocorrelation. After this, we can select a noise model function that fits the empirical data; in our case, this is an exponentially decreasing function:

(8) f(r|A,λ)=A·exp(−λr)

where $r=\sqrt{x^{2}+y^{2}}$ is the pixel lag, *A* is the noise amplitude (i.e., variance), and λ is the correlation length parameter. Figure 4 shows the 1D empirical autocorrelations along with the fitted exponential functions and the empirical cross-correlation. We can observe that the autocorrelation approaches zero around the 10th-pixel lag, indicating pixel correlation within this range. The parameters of fitted functions for the real and imaginary parts, also depicted in Figure 4, are approximately the same—meaning that we can model them with the same function. Note that the standard deviation of the noise ($\sigma =\sqrt{A}$ derived from Equation (5)) is approximately 0.7, which aligns with the parameters of the previously attained normal pixel intensity distribution function shown in Figure 2. Using this autocorrelation function, we can model and synthesize noise with both the required normal pixel intensity distribution and spatial correlation properties.

We should mention that an exponential ACF leads to a Lorentzian power spectral density (PSD), and this does not imply a strict 1/f or $1/f^{2}$ noise behavior. Instead, it displays a more complex dependency on the frequency, which might be close to 1/f-like pink noise.

**Figure 5:**
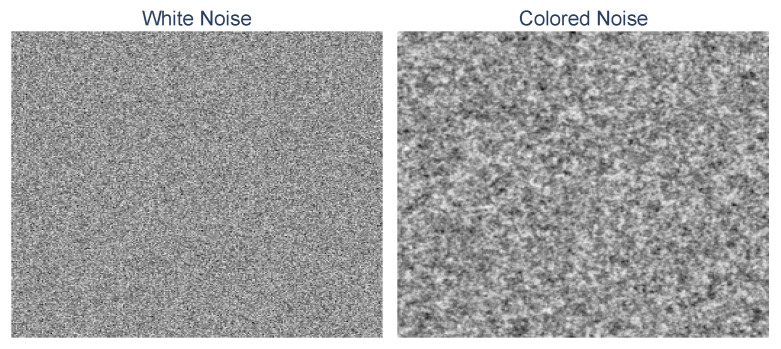
Example of generated white noise and exponential colored noise.

**Figure 6:**
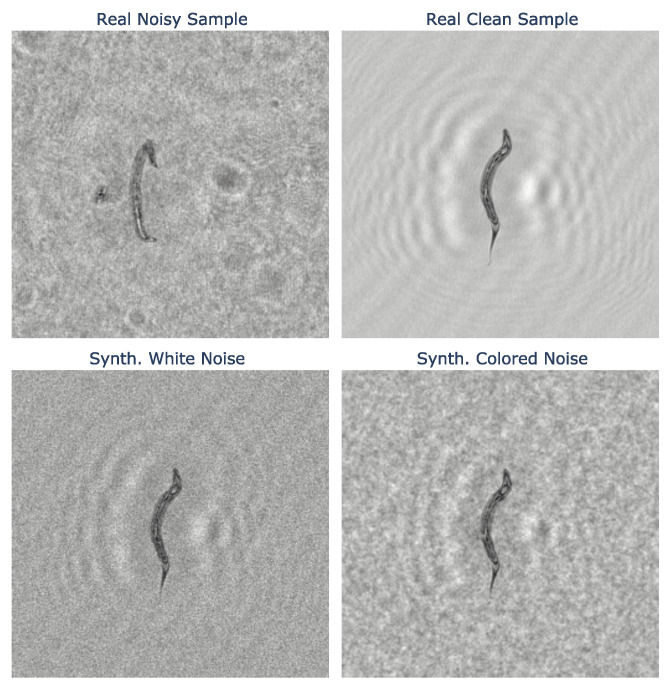
Example of white noise and exponential colored noise added to a clean sample to augment a noisy reconstructed hologram.

Figure 5 illustrates the visual difference between random white noise and exponentially correlated random colored noise. Figure 6 exemplifies the process of augmenting a noisy reconstructed hologram using a clean sample and compares the outcomes to a real-world noisy sample. We can observe from the figure that the sample synthesized using colored noise appears more realistic than the one synthesized using uncorrelated white noise. The illustrations were generated using the fitted noise model parameters.

### 3.2. Neural Network Training

In this section, we investigate the effect of noise augmentation on the performance of neural networks in the case of classifying clean and noisy reconstructed hologram samples.

**Figure 7:**
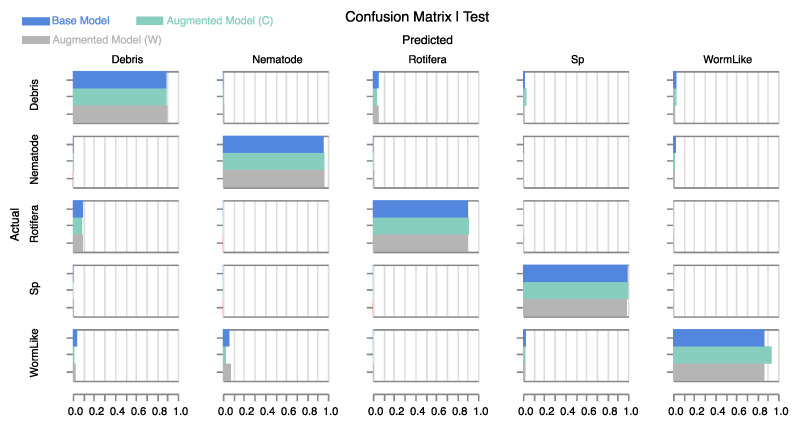
Normalized confusion matrix for the base and augmented models (W: white noise, C: colored noise) on **clean test samples.** The performance on clean samples is not degraded in the case of augmented models. Color noise augmentation slightly improves classification performance on clean samples, especially for the *WormLike* class.

First, we confirmed our hypothesis that adding noise during training does not significantly affect the classification of clean samples. Multiple calculated metrics on the test set—shown in Table 1—support this idea. Figure 7 displays the confusion matrix of the *base model* and the *augmented models*. We can see that the performance is not only retained, but it is even slightly improved in the case of the color-augmented model—which indicates that the application of realistic noise augmentation can be beneficial to the classification of even the standard clean samples. The improvement is most prominent in the *WormLike* class, where the model is less likely to misclassify worm-like objects as nematodes. In our clean dataset, worm-like holograms generally exhibit higher levels of background noise compared with nematode holograms, which are cleaner. This could explain why the base model is more likely to misclassify less noisy worm-like objects as nematodes. On the contrary, applying white noise augmentation does not result in improvement, suggesting that the application of a realistic noise model is important to attain beneficial effects.

**Figure 8:**
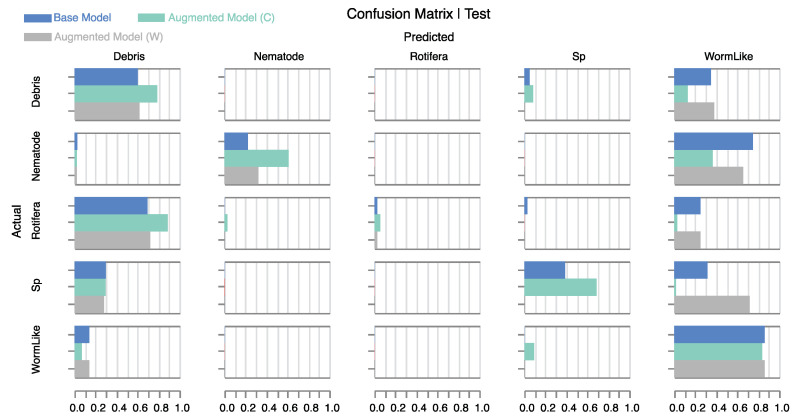
Normalized confusion matrix for the base and augmented models (W: white noise, C: colored noise) on **noisy test samples.** The colored noise model significantly improves classification performance compared with the other models. The white noise model improves nematode classification but completely misses the *Sp* class.

In Table 2, performance metrics are shown for the same models but for the noisy test dataset. The overall accuracy of the color-augmented model is improved by over 20% compared with the base model. In contrast, the white-augmented model shows a notable decline in performance. For a more detailed analysis of model behavior, refer to the confusion matrix in Figure 8. The color-augmented model outperforms the other models across all individual classes. Specifically, it more than doubles the correct nematode predictions compared with the base model. Although distinguishing rotifera from debris is particularly challenging in noisy holograms—reflected in the low count of true positives—the color-augmented model still manages to identify twice as many. Additionally, the model significantly improves the classification of debris and small particles, while substantially reducing the number of false positives for worm-like objects. The tendency of the base model to predict numerous false positives for the *WormLike* category could be attributed to the inherently higher noise levels found in the clean worm-like samples within our database. This suggests that the base model has incorrectly learned to use background noise as a characteristic feature. As a result, it has a propensity to categorize any noisy image as belonging to the class—specifically the *WormLike* class, in our case—that exhibited higher noise levels during training. Importantly, our color noise augmentation method addresses this issue by equalizing background noise across different classes, allowing the model to focus solely on object morphology for classification.

The white noise-augmented model exceeds the baseline only in the classification of nematodes. Strikingly, it fails to correctly classify any of the small particles, completely undermining that category. This serves as a cautionary note that applying unrealistic noise models can not only be ineffective but also severely compromise model performance.

These results strongly underscore the central argument of this article: incorporating a realistic noise augmentation model into the training process has a transformative impact on the model’s performance. Specifically, when faced with unseen noisy samples, a model trained with our realistic noise augmentation significantly outperforms models that were trained without it or with a less accurate noise model.

Additionally, it is worth noting that introducing our realistic noise augmentation into the training process did not lead to any increase in computational burden. For all experiments, the training time remained consistent at approximately 1 h and 30 min when run on an NVIDIA GeForce GTX 1060. In both the augmented and non-augmented cases, early-stopping criteria were met around the 50th epoch, indicating that the noise augmentation did not prolong the training duration.

## 4. Conclusions

The examined noise (sometimes present in our reconstructed holograms) is a colored (correlated) noise rather than simple white noise that requires appropriate modeling. The assumptions for deriving our colored noise model were that the pixel density distribution of the noise is normal, the 2D empirical autocorrelation of the noise is isotropic, and the real and imaginary parts of the complex reconstructed hologram noise are independent. As shown in the preceding section, these assumptions approximately hold for our data.

In the case of standard clean inputs, incorporating noise augmentation in training not only preserves but even slightly improves the model’s classification ability. This may be because the clean database itself is not entirely noise-free and because the augmentation process forces the model to learn better feature representations. Consequently, the neural network becomes proficient at classifying both clean and noisy images at the same time while focusing more on object attributes and less on background noise.

The base model (without noise augmentation) was misled by quality-degraded input images and tended to classify noisy images into one class. Applying traditional Gaussian white noise augmentation increased model accuracy in the case of nematode classification, but completely malfunctioned in small-particle detection. The proposed colored noise model produces augmented images that more closely resemble the actual noisy data, resulting in a significant accuracy increase of more than 20% compared with the base model in the case of noisy test data.

From the results, we can conclude that the application of a realistic noise model is beneficial to make the system more robust for quality-degraded input images and to force the model to learn better feature representations by disregarding background noise.

Moreover, incorporating noise augmentation into training did not extend the training time in our experiments, suggesting that we can enhance model performance without additional computational burden and costs.

## 5. Summary

In this paper, we addressed the challenge of inconsistent image quality during extended recording periods, a factor that can compromise the performance of neural networks. We explored this challenge in the specific context of classifying (unseen) quality-degraded reconstructed holograms. Our proposed approach requires only a minimal set of background noise images to construct a colored noise model which is then used to augment clean training data, enabling the neural network to perform more reliably when faced with quality-degraded inputs.

Our data analysis revealed the presence of exponentially correlated colored noise, contrasting the simpler white noise. We fitted noise models to empirical data and then illustrated noisy sample augmentation. Visually, the colored noise model closely mimicked real-world noisy samples, unlike simple white noise perturbations.

We demonstrated that our colored noise augmentation not only preserves but also enhances neural network performance on standard clean samples. This improvement is attributed to the network’s focus on relevant features, ignoring class-specific background noise (moreover, the clean dataset itself might contain minor noise influences).

A neural network trained solely on clean data dropped to 47% accuracy on degraded samples. On the contrary, our color-augmented noise model achieved an accuracy rate of 71%. Basic Gaussian white noise augmentation, while benefiting nematode classification, disrupted small-particle identification, highlighting our model’s advantage and the need for realistic noise models for significant performance improvements.

Though specific to a certain problem, our method is broadly applicable to scenarios with intricate colored noise in images. With a few background noise images, we can determine the autocorrelation function representing the noise, fit it to empirical data, and use it for training augmentation. This approach, applicable across neural networks, does not extend training time and improves system robustness to noise, while maintaining or enhancing performance on high-quality inputs.

## Figures and Tables

**Table 1:** Performance metrics for the base and augmented models on the **clean test dataset**. The performance of the augmented models is essentially the same for clean samples; the white noise model is a little worse, while the one with colored noise slightly outperforms the base model.

Model	Accuracy	Precision	Recall	F1 Score
Base model	0.931	0.929	0.931	0.93
Augmented model (W)	0.928	0.928	0.928	0.928
Augmented model (C)	**0.935**	**0.933**	**0.935**	**0.933**

**Table 2:** Performance metrics for the base model and models trained with augmented datasets utilizing white noise (W) and colored noise (C) on the **noisy test dataset**. The performance of the colored model is superior to that of the white and base models.

Model	Accuracy	Precision	Recall	F1 Score
Base model	0.47	0.68	0.42	0.52
Augmented model (W)	0.26	0.64	0.37	0.46
Augmented model (C)	**0.71**	0.67	**0.60**	**0.63**

## Data Availability

The underlying data supporting the results presented in this paper are not publicly available due to commercial and intellectual property considerations. However, interested researchers may request access to the data directly from the authors.

## Abbreviations

The following abbreviations are used in this manuscript:

ACF	Autocorrelation function
NN	Neural Network
DHM	Digital Holographic Microscope
